# Tracking cashew economically important diseases in the West African region using metagenomics

**DOI:** 10.3389/fpls.2015.00482

**Published:** 2015-06-30

**Authors:** Filipa Monteiro, Maria M. Romeiras, Andreia Figueiredo, Mónica Sebastiana, Aladje Baldé, Luís Catarino, Dora Batista

**Affiliations:** ^1^BioISI - Biosystems and Integrative Sciences Institute, Faculdade de Ciências, Universidade de Lisboa, Lisboa, Portugal; ^2^Tropical Research Institute, Lisboa, Portugal; ^3^Instituto Piaget da Guiné-Bissau, Bissau, Guiné-Bissau; ^4^Centre in Biodiversity and Genetic Resources, Universidade do Porto, Vairão, Portugal; ^5^cE3c - Centre for Ecology, Evolution and Environmental Changes,Faculdade de Ciências, Universidade de Lisboa, Lisboa, Portugal

**Keywords:** Africa, *Anacardium occidentale*, fungal diseases, Guinea-Bissau, mycobiome, next generation sequencing

## Abstract

During the last decades, agricultural land-uses in West Africa were marked by dramatic shifts in the coverage of individual crops. Nowadays, cashew (*Anacardium occidentale* L.) is one of the most export-oriented horticulture crops, notably in Guinea-Bissau. Relying heavily on agriculture to increase their income, developing countries have been following a strong trend of moving on from traditional farming systems toward commercial production. Emerging infectious diseases, driven either by adaptation to local conditions or inadvertent importation of plant pathogens, are able to cause tremendous cashew production losses, with economic and social impact of which, in developing countries is often underestimated. Presently, plant genomics with metagenomics as an emergent tool, presents an enormous potential to better characterize diseases by providing extensive knowledge on plant pathogens at a large scale. In this perspective, we address metagenomics as a promising genomic tool to identify cashew fungal associated diseases as well as to discriminate the causal pathogens, aiming at obtaining tools to help design effective strategies for disease control and thus promote the sustainable production of cashew in West African Region.

## Cashew as an Export-Oriented Horticulture Crop in West Africa

During the past decades, agricultural land-use changes in West Africa were marked by an initial increase in total cropped area, followed by dramatic shifts in the coverage of individual crops. Among the crops that had a more recent expansion in the West Africa Region, stands out the cashew. Cashew (*Anacardium occidentale* L.) is a tropical evergreen tree, which belongs to the Anacardiaceae family that also comprises other economically important crops, including mangos (*Mangifera indica* L.) and pistachios (*Pistacia vera* L.; Pell, 2004).

**FIGURE 1:**
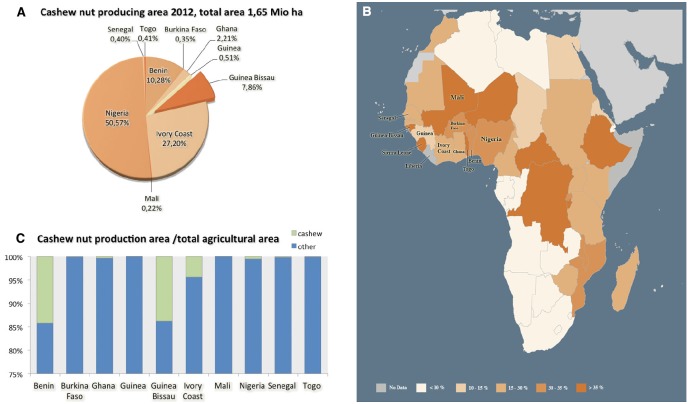
**Cashew production with detailed data for West African countries: (A) Cashew nut producing area against the total amount in Millions tons produced in 2012 (1,65 Mio ha); (B) Map of the share of agriculture in gross domestic product (GDP %, 2000–2010; Adapted with permission from Food and Agricultural Organization of the United Nations, 2012b); colors indicate different percentage as indicated in the map legend; (C) Total agriculture area against the cashew nut area per country.** Data retrieved from FAOSTAT Database on 29 January 2015 for cashew nut with shell in West African countries.

The cashew is native to Central and South America with Eastern Brazil as its primary center of diversity, and was introduced in Africa during the second half of the sixteenth century (Salam and Peter, 2010). Cashew is nowadays an important export-oriented horticulture crop, being produced under intensive cultivation regimes in several tropical regions (Aliyu, 2008). According to Food and Agricultural Organization of the United Nations (2012a), the annual total world production of cashew nuts is approaching one million MT, being Vietnam (30%) and Nigeria (21%) the major producers, followed by Brazil, with further significant yields in West African countries, namely in Ivory Coast, Benin and Guinea-Bissau (Figure 1A). Indeed, in several African countries such as Guinea-Bissau the share of agriculture in gross domestic product (GDP) is very important since it represented over 35% of gross national product (GNP) in 2010 (Figure 1B), thus reinforcing the role of agriculture on least developed countries (LDCs; World Bank, 2010; Food and Agricultural Organization of the United Nations, 2012b). Cashew is by far the most important cash crop grown in Guinea-Bissau, and it is estimated that cashew orchards cover about 210,000 ha (Kyle, 2009; Figure 1C), with a tendency of keep growing. In the last three decades, the cashew sector has acquired an enormous significance in Guinea-Bissau’s economy, both in terms of governmental revenues and on social impact, involving in some way more than 85% of the rural population (Kyle, 2009). This over-dependence on a single crop involves risks to the national economy. Since no plant breeding strategies or suitable husbandry practices have been implemented, challenges to cashew sustainable production in Guinea-Bissau are even more pressing and should thus be carefully considered.

Fungal diseases represent one of the major threats to crop-based global economy and food safety. Emerging infectious diseases, caused either by pathogens occurring on a novel host and/or in a new or increased geographical area, have been arising at an increasing rate on a wide range of host plants as a consequence of the domestication of ecosystems or expanding global trade (Gladieux et al., 2011; Silva et al., 2012a). In developing agro-ecosystems shaped by large monocultures and genetically uniform hosts, disease emergence can be a worst-case scenario, which often results in devastating epidemics (Desprez-Loustau et al., 2007). Considering further the inadequate functioning of the agricultural extension in Guinea-Bissau and some worrying signs of localized symptomatic trees, a disease outbreak on cashew orchards is likely to become a serious problem in a near future with dramatic associated losses for both economic and social sectors (Catarino et al., 2015). The historical and potential impacts of invasive pathogens in agriculture call for urgent intervention measures, which should begin by a complete assessment of the diseases that affect cashew orchards in order to plan disease control.

## Linking the Emergence of Fungal Diseases with Cashew Orchards

Cashew is susceptible to over 10 diseases caused by fungi (Cardoso et al., 2013). Anthracnose foliar blight and fruit rot (*Colletotrichum gloeosporioides* Penz. & Sacc.) and gummosis of twigs and trunk [*Lasiodiplodia theobromae* (Pat.) Griffon & Maubl.] are often considered the most relevant diseases causing severe damages across cashew producing countries (Ghini et al., 2011). In Brazil, although an increase in gummosis severity has been reported in all northeastern producing states (Cardoso et al., 2004; Cysne et al., 2010; Moreira et al., 2013), anthracnose is by far the most important disease in the field, leading to significant yield losses (e.g., Freire et al., 2002; Araújo, 2013). Other foliar infections, namely black mold [*Pilgeriella anacardii* (Bat., J.L. Bezerra, Castr. & Matta) Arx & E. Müll.] and powdery mildew (*Oidium anacardii* F. Noack), also occur but with almost negligible consequences in cashew orchards (Freire et al., 2002; Ghini et al., 2011).

Most epidemiological studies on these cashew-affecting diseases have been performed in Brazil, where the occurrence of anthracnose was first reported in 1948 (Rossetti, 1948). The causal agent, *C. gloeosporioides*, is a common pathogen of other tropical fruit plants (Figueiredo et al., 2012), highly variable in cultural and morphological characters, and in pathogenicity (Freire and Cardoso, 2003). The pathogen can infect leaves, twigs, inflorescences, young apples and fruits, and symptoms include sunken subcircular or angular lesions that produce erumpent, mucilaginous, orange spore masses in favorable environments (Lopez and Lucas, 2010). In severe cases, leaves and fruitlets become totally blighted and drop (Freire et al., 2002). Precipitation, humidity and temperature are key factors for the fungus dispersal and infection. *C. gloeosporioides* survives within remains of infected tissues on the soil, but spore dispersal by rain represents the most relevant source of dissemination (Cardoso and Viana, 2011). The spores are bonded and involved in a mucilaginous layer which protects them from dissection in dry weather until subsequent rains splashes them further away. The impact of raindrops spreads the spores to a variable range of distances, which may be further enhanced by the wind (Ntahimpera et al., 1999). Thus, during the rainy periods the disease reaches its highest severity, spreading quickly inside and between plants. Temperatures ranging from 22 to 28°C and at least 10 h of saturation are excellent conditions for the infection (Freire et al., 2002). When such conditions of high humidity and rainfall prevail during the flowering stage until the beginning of cashew nut setting, apple and kernel quality are drastically affected and production losses are even more significant (Cardoso and Viana, 2011; Uaciquete et al., 2013).

Presently, anthracnose is highly prevalent in all cashew-growing regions and provinces of Brazil and Mozambique (Cardoso and Viana, 2011; Uaciquete et al., 2013). Despite the incremental nature of *C. gloeosporioides*’s splash dispersal through time, its overall capacity is still quite low to account for the large extent of anthracnose dissemination found in these major cashew producing countries. Other phenomena, such as animal and anthropogenic dispersal, are likely to have significantly contributed, mostly due to agricultural practices and through introduction of infected plant materials. Moreover, the evidence for anthracnose cross-infection in cashew from other susceptible plants cultivated in close proximity under mixed cropping systems (Lopez and Lucas, 2010; Lakshmi et al., 2011), calls for prudence and concern in managing disease control for cashew production.

In contrast, the general panorama in West African countries is largely unknown and few studies are available. In Nigeria, studies were conducted on the incidence and impact of these diseases, reporting the identification of *C. gloeosporioides* among other fungi (Otuonye et al., 2014), and evidences of cashew gummosis (e.g., Adejumo, 2005; Adeniyi et al., 2011). Particularly in Guinea-Bissau, a growing detection of dying trees is becoming alarming and no information is available about which and how fungal diseases are affecting cashew production. Given this scenario, the experience with diseases in other cashew producing countries (e.g., Mozambique and Brazil), particularly anthracnose, demonstrate that an early action is of the utmost importance in controlling the disease and limiting production losses.

**FIGURE 2:**
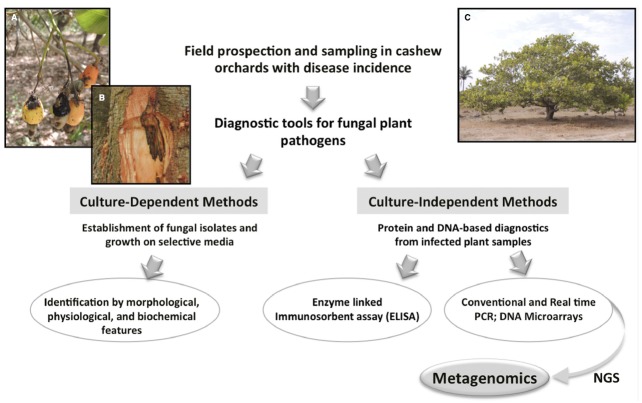
**Workflow approach proposed for cashew disease characterization based on metagenomics, illustrating in parallel different methods allowing fungal pathogen detection and identification.** Culture-dependent methods rely on pathogen isolation and culture on selective media for species identification based on morphocultural characters. Culture-independent methods allow the direct analysis of infected plant samples for pathogen detection by targeting specific proteins (ELISA) or DNA sequences (e.g., PCR, microarrays), and thus require previous knowledge of pathogen sequence data. Besides detecting the presence or absence of the pathogen, some DNA-based methods are also quantitative (real-time PCR) and permit multiplexing (microarrays), enabling the detection of multiple targets. Among the molecular methods, metagenomics is highlighted as a promising tool to perform a large-scale analysis of the mycobiota associated with diseased cashew plants. Evidences of anthracnose symptoms in cashew nuts and apples **(A)** and of gummosis in the trunk **(B)** collected during field surveys on cashew orchards in the North region of Guinea-Bissau **(C)** (Photos by L. Catarino).

Recently, an initial phytosanitary survey of cashew orchards in Guinea-Bissau was undertaken in the framework of the international project “*Cashew in West Africa: socio-economic and environmental challenges of an expanding cash crop*”. This preliminary field prospection across the Northern and Eastern regions, allowed us to identify symptoms recognizable as anthracnose and gummosis (see Figure 2), although other fungal-associated diseases may probably remain to be uncovered. Indeed, it is not uncommon to find several distinct fungal pathogenic species in diseased plants (Desprez-Loustau et al., 2007). Recently, a new pathogenic fungus (*Cryptosporiopsis* spp.) causing cashew blight disease was reported in Tanzania (Dominic et al., 2014). Considering the scarce information on cashew diseases, a broad approach that could accurately identify and analyze mycobiota responsible for such disorders would contribute to design effective control measures. Moreover, the identification of some pathogens is not completely straightforward. For instance, *C. gloeosporioides*, ascribed to anthracnose, represents in fact a species complex of cosmopolitan pathogens with an exceptionally broad host range (Silva et al., 2012b), which has long presented confusing boundaries. Systematics within the complex was much improved in the last years particularly since the designation of an epitype specimen (Cannon et al., 2008) and the development of phylogenetic studies based on multi-locus analyses. Upon the latest revision by Weir et al. (2012), this species aggregate is now considered to consist of at least 22 species traditionally referred to as *C. gloeosporioides*, including *C. gloeosporioides sensu sricto (s.s.)*, many of which cannot be reliably distinguished using ITS, the official barcoding gene for fungi. Presently, additional sequencing of secondary barcodes [i.e., glyceraldehyde-3-phosphate dehydrogenase (*GAPDH*), glutamine synthetase (*GS*) and *ApMAT* intergenic region] allows a further species identification within the *C. gloeosporioides* complex (Silva et al., 2012b; Weir et al., 2012). Under the recently more accurate molecular-based taxonomy, *C. gloeosporioides s.s.* is now known to be much less common in the environment than previously thought (Crouch et al., 2014). Cashew anthracnose may fall into this challenging issue, encompassing more than one species or a distinct species from the ex-epitype strain, since pathogen molecular identifications performed up to now were mostly based on ITS (e.g., Figueiredo et al., 2012; Uaciquete et al., 2013). Accurate identification of causal agents is crucial to improve biosecurity and disease control, since misdiagnosis can have serious negative consequences affecting unequivocally the specific control actions implemented.

## Metagenomics as a Promising Approach to Track Cashew Disease-Associated Pathogens

Fungi are often difficult to detect without a concerted effort using special cultural methods and molecular tools (Desprez-Loustau et al., 2007). Even so, typical confusing morphological characters of fungal pathogens have been pushing pathogen identification to move fast from relying on traditional cultural and morphology-based methods to modern molecular-based methods (Figure 2). Culture-dependent approaches rely on the ability of the microorganisms to grow on artificial media, are very time-consuming and require extensive taxonomical expertise. These limitations have been surpassed by the use of immunological assays (ELISA) and DNA-based technologies, which additionally allow testing asymptomatically infected plant material. Among the more recently applied diagnostic tools, real time PCR and microarray techniques revealed their potential for the reliable and fast identification of plant pathogens (e.g., Boonham et al., 2008; McLoughlin, 2011), but prior sequence data knowledge of the target pathogens is required, leaving out uncharacterized pathogens. PCR-based methods have become important tools to detect and/or quantify specific pathogens, yet fail to identify many strains/races and distinguish taxonomically close pathogen species. In this context, next generation sequencing technologies (NGS) have the potential to circumvent such methodological limitations, opening new possibilities to explore uncharacterized plant disease systems. Some of these methods used in plant pathogen diagnostics are summarized in Figure 2.

Metagenomics, in particular, has been gaining relevance by the remarkable advances that has provided on the survey and characterization of whole microbial communities contained in specific environmental samples (Unterseher et al., 2011; Bragg and Tyson, 2014). This powerful genomic approach is based on the analysis of collective microbial genomes, regardless of their ability to be cultured in the laboratory, to understand the genetic diversity, population structure, and ecological roles within the communities probed (e.g., for reviews, see Guttman et al., 2014; Melcher et al., 2014). Metagenomics takes advantage of NGS for the large-scale study of microbial populations by analyzing the whole nucleotide sequence content of a sample. Thus, valuable outcomes from the use of metagenomics are also expected beyond the more common focus on soil, water, or extreme environments (Cuadros-Orellana et al., 2013). Only recently metagenomics emerged as a novel tool for studying pathogenic microbe–plant interactions (Faure et al., 2011; Knief, 2014), holding great promise to identify the extant pathogen range in uncharacterized plant disease systems. In a metagenomic approach to diagnose plant pathogens, nucleotide sequences from an infected plant, including sequences from any pathogen present, can potentially be sequenced and analyzed. Important contributions to this field were provided by recent metagenomic studies, particularly on bacterial and viral plant pathogens. For instance, metagenomics was used to validate and characterize the putative causal agent of the citrus disease Huanglongbing (HLB), also known as citrus greening, which is a devastating disease associated with the presence of three unculturable bacteria members of the genus “*Candidatus Liberibacter*” (Duan et al., 2009; Tyler et al., 2009). These studies provided an estimation of the bacterial cell density per plant cell and the complete genome of the confirmed pathogen “*Ca. L. asiaticus*.” Also, analysis of metagenomic data from citrus leprosis symptomatic leaves in Colombia led to the identification of a novel virus of the genus *Cilevirus* (Roy et al., 2013). Similarly, Rwahnih et al. (2009) reported the presence of a novel virus within the multiple virus infection associated with decline symptoms of Syrah grapevines. Based on the complete genome sequence of the novel virus obtained in this study, an RT-PCR test was developed to further analyze its field distribution in California.

Presently, with the decreasing costs in sequencing due to faster and more powerful high-throughput methods and the increasing level of sample sequence coverage, metagenomics is becoming an effective method for studying plant pathogens. The ability to analyze NGS data, while constantly growing through the pursue of improved computational resources and dataset assembly strategies, is still however a major bottleneck in achieving many of the goals of metagenomic studies (Melcher et al., 2014).

Depending on the aim of the project, different metagenomic strategies can be delineated taking into account the most suitable sequencing platform, downstream analyses and bioinformatics tools, within a whole-genome versus a targeted amplicon sequencing approach (Knief, 2014). Targeted sequencing metagenomics studies rely on the use of universal primers for the amplification and subsequent next-generation sequencing of DNA from microbial communities, such as the 16S rRNA gene for bacteria or the ITS for fungi (Bokulich et al., 2014; Knief, 2014). After bioinformatics analysis and comparison with databases, the whole microbial composition of the sample can be identified.

Considering the challenging and poorly known scenario of cashew fungal diseases in Guinea-Bissau, targeted mycobiome metagenomics constitutes an ideal approach for profiling the associated fungal community and comparing its composition in different healthy and symptomatic samples. Following this strategy, global fungal diversity can be assessed by next-generation sequencing of the ITS region, but in cashew’s case, for which anthracnose is one of the most prominent diseases, combinatorial sequencing of secondary barcodes (*GADPH*, *GS*, and *ApMAT*) is recommended for further species level identification within the *C. gloeosporioides* complex (Weir et al., 2012). The analysis of the metagenomic data will generate an unprecedented amount of information allowing a detailed characterization of the mycobiota associated with diseased cashew plants. This will enable accurate identification of the causal pathogens along with categorization of main incident diseases, but also can uncover previously unknown/undetermined pathogens or unculturable species, and discriminate other fungal species playing specific ecological roles. Moreover, within a biogeographic framework, the pathogen metagenomic data can be further explored in population and evolutionary genomic analyses to trace patterns of migration/dispersal, gene flow and phylogeographical structure, estimate evolutionary potential and infer levels of adaptive evolution. A better understanding of cashew’s pathogen population structure and dynamics will increase the basis of recommendations for the management of diseases, influencing breeding programs.

In the light of the increasing need to control the emergence and spread of cashew diseases in Guinea-Bissau, the pursue of such a genomic approach would boost our insight into the extant pathogen populations as a tool to help developing improved disease management strategies, and thus promote the sustainable production of cashew in West Africa.

### Conflict of Interest Statement

The authors declare that the research was conducted in the absence of any commercial or financial relationships that could be construed as a potential conflict of interest.
